# Radiotherapy plus anti-PD1 versus radiotherapy for hepatic toxicity in patients with hepatocellular carcinoma

**DOI:** 10.1186/s13014-023-02309-1

**Published:** 2023-08-04

**Authors:** Rui-Jun Zhang, Hong-Mei Zhou, Hai-Yan Lu, Hong-Ping Yu, Wei-Zhong Tang, Mo-Qin Qiu, Liu-Ying Yan, Mei-Ying Long, Ting-Shi Su, Bang-De Xiang, Mei-Ling He, Xiao-Ting Wang, Shi-Xiong Liang, Jian-Xu Li

**Affiliations:** 1 Department of Radiation Oncology, Guangxi Medical University Cancer Hospital, Nanning, 530021 China; 2 Department of Research, Guangxi Medical University Cancer Hospital, Nanning, 530021 China; 3 Division of Colorectal & Anal Surgery, Department of Gastrointestinal Surgery, Guangxi Medical University Cancer Hospital, Nanning, 530021 China; 4 Department of Respiratory Oncology, Guangxi Medical University Cancer Hospital, Nanning, 530021 China; 5 Department of General Affairs, Guangxi Medical University Cancer Hospital, Nanning, 530021 China; 6 School of Public Health, Guangxi Medical University, Nanning, 530021 China; 7 Department of Hepatobiliary Surgery, Guangxi Medical University Cancer Hospital, Nanning, 530021 China

**Keywords:** Anti-PD1, Hepatocellular carcinoma, Propensity score matching, Radiation-induced liver disease

## Abstract

**Purpose:**

In this study, we aimed to compare the radiation-induced hepatic toxicity (RIHT) outcomes of radiotherapy (RT) plus antibodies against programmed cell death protein 1 (anti-PD1) versus RT alone in patients with hepatocellular carcinoma (HCC), evaluate prognostic factors of non-classic radiation-induced liver disease (ncRILD), and establish a nomogram for predicting the probability of ncRILD.

**Patients and methods:**

Patients with unresectable HCC treated with RT and anti-PD1 (RT + PD1, n = 30) or RT alone (n = 66) were enrolled retrospectively. Patients (n = 30) in each group were placed in a matched cohort using propensity score matching (PSM). Treatment-related hepatotoxicity was evaluated and analyzed before and after PSM. The prognostic factors affecting ncRILD were identified by univariable logistic analysis and Spearman’s rank test in the matched cohort to generate a nomogram.

**Results:**

There were no differences in RIHT except for increased aspartate aminotransferase (AST) ≥ grade 1 and increased total bilirubin ≥ grade 1 between the two groups before PSM. After PSM, AST ≥ grade 1 occurred more frequently in the RT + PD1 group (p = 0.020), and there were no significant differences in other hepatotoxicity metrics between the two groups. In the matched cohort, V25, tumor number, age, and prothrombin time (PT) were the optimal prognostic factors for ncRILD modeling. A nomogram revealed a good predictive performance (area under the curve = 0.82).

**Conclusions:**

The incidence of RIHT in patients with HCC treated with RT + PD1 was acceptable and similar to that of RT treatment. The nomogram based on V25, tumor number, age, and PT robustly predicted the probability of ncRILD.

**Supplementary Information:**

The online version contains supplementary material available at 10.1186/s13014-023-02309-1.

## Introduction

Hepatocellular carcinoma (HCC) is a major global health problem, and its incidence is currently rising in most countries [1]. The primary treatment options for early-stage HCC are surgery, radiofrequency ablation, and orthotopic liver transplantation [2–4]. Unfortunately, most patients with HCC are diagnosed with advanced disease and a poor prognosis [5]. Currently, the first-line molecular-targeted therapy for unresectable HCC includes treatment with sorafenib and lenvatinib, and the overall survival of patients with HCC is still unsatisfactory [6]. Antibodies against programmed cell death protein 1 (anti-PD1) have yielded promising results for advanced HCC [7]. In the IMbrave150 study, atezolizumab plus bevacizumab led to significant survival benefits; however, the combination incurs a high cost and results in 56.5% grade ≥ 3 TRAEs [8].

With advancements in radiotherapy (RT) technologies, including intensity-modulated radiation therapy (IMRT), an increasing number of patients with HCC have achieved good disease control after radiotherapy (RT) [9]. Radiotherapy for patients with liver cancer has been recommended by the National Comprehensive Cancer Network as a standard treatment method [10]. RT can potentiate tumor immunity and enhance antitumor effects in combination with immunotherapy [11]. A case series involving five patients with unresectable HCC treated with stereotactic body radiotherapy (SBRT) followed by anti-PD1 reported a 100% response rate to treatment and a median PFS of 14.9 months [12]. In a phase II trial, the combination of RT with camrelizumab (an anti-PD1) for patients with unresectable HCC showed promising efficacy and acceptable safety profile, with 52.4% of patients achieving an objective response [13]. Combined SBRT and immunotherapy resulted in significantly superior survival and less toxicity compared with transcatheter arterial chemoembolization (TACE) [14]. The combination of RT with anti-PD1 may, therefore, be a novel therapeutic strategy for HCC.

Radiation-induced hepatic toxicity (RIHT) is a common dose-limiting factor in the use of RT for HCC, in which the radiation-induced liver disease (RILD) is described as severe RIHT [15, 16]. Multiple studies have shown that the adverse reactions to anti-PD1 include abnormal hepatic function, including elevated transaminase and/or elevated total bilirubin [17–19]. However, it is unclear whether the combination of radiotherapy with anti-PD1 increases the incidence of RIHT. This study aimed to compare the severity of RIHT between RT combined with anti-PD1 (RT + PD1) versus RT alone for HCC. In addition, prognostic factors for RILD were investigated.

## Materials and methods

### Patients

**Fig. 1:**
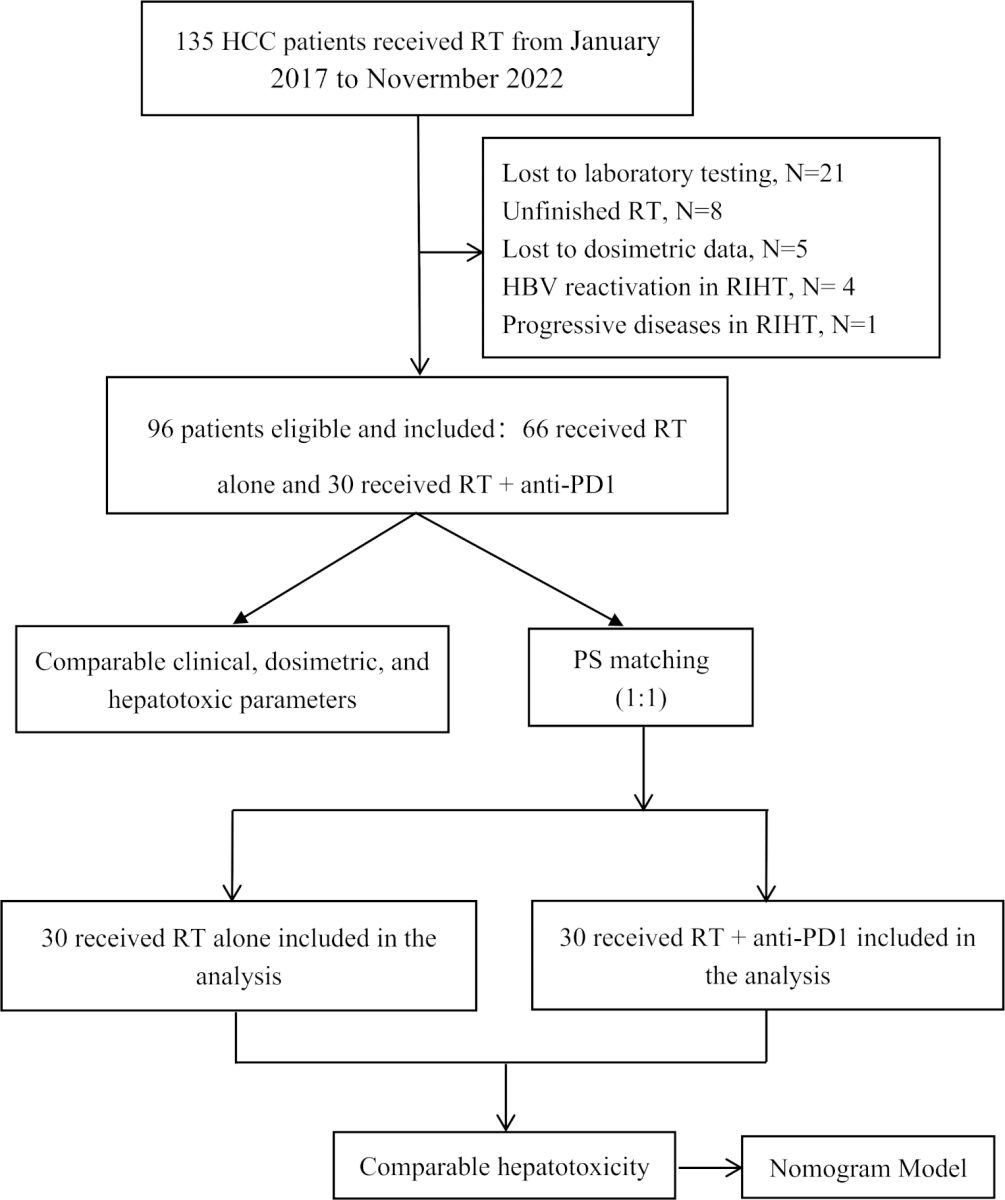
Study flow diagram. Anti-PD1, antibodies against programmed cell death protein 1; HBV, hepatitis B virus; HCC, hepatocellular carcinoma; RIHT, radiation-induced hepatic toxicity; RT, radiotherapy

All patients with HCC undergoing radiotherapy were screened between January 2017 and November 2022. The patients were diagnosed with HCC histologically and/or radiologically based on the guidelines of the American Association for the Study of Liver Diseases [20] and staged according to the Barcelona Clinic Liver Cancer (BCLC) system [21]. The general inclusion criteria for the study were as follows: [1] Patients with Child–Pugh (CP) class A or B and an Eastern Cooperative Oncology Group performance score of 0–2; [2] were not combined with intrahepatic cholangiocarcinoma; [3] had not received concurrent targeted therapy; [4] had not received surgery and ablation therapy between one month before the first fraction of radiotherapy and three months after the last fraction; [5] recovery of all hepatotoxic conditions of patients to grade 1 or less before the first fraction of radiotherapy in those who received prior interventional therapy; [6] patients without interventional therapy during RT and three months after the last fraction; [7] availability of dose–volume histogram (DVH) dosimetric parameters and RIHT-relevant data. After applying these criteria, 135 patients were registered; of these, 39 were excluded as shown in Fig. 1. Total of 96 patients were ultimately enrolled in this study, including 30 patients treated with RT plus anti-PD1 (RT + PD1 group) and 66 patients treated with RT alone (RT group) (Fig. 1). Finally, 30 patients in each group were included in the matched cohort. Ethical approval was obtained from the Guangxi Medical University Cancer Hospital (LW2022112).

### Radiotherapy protocol

Contrast-enhanced computed tomography (CT) scans for RT planning were performed at 2.5–5 mm slice thickness under spontaneous breathing in the supine position. The gross tumor volume (GTV) was defined as the size of the intrahepatic tumor that was enhanced in the arterial phase. The magnetic resonance and CT images were then fused to better sketch the GTV. To compensate for organ motion and setup error, the planning target volume (PTV) comprised the GTV plus a 5–10 mm margin in all directions. All target volumes and organs at risk were delineated using the MIM 6.8 system (MIM, USA). The plans were designed using IMRT or volumetric-modulated arc therapy (VMAT). Based on this plan, the Pinnacle 3 system (Philips, Netherlands) or Monaco treatment planning system (version 5.1) was generated. A 6 MV X-ray (ELEKTA Versa-HD or ELEKTA Synergy, Sweden) linear accelerator was used.

The fractionated radiation doses were chosen based on the principles of 2 to 6 Gy/fraction. The patients received a median total IMRT dose of 51.0 Gy (47.5–60.0 Gy) with a median of 3.0 Gy (2.4–4.0 Gy) per fraction for a median of 20 (15-20) fractions administered five days a week. The organs at risk (OARs) were well protected when the DVH analysis was performed to evaluate the radiotherapy plan. For the liver, the mean dose to the normal liver (Dmean) was less than 21 Gy. For the kidneys, V15 was < 1/3 volume. For the spinal cord, Dmax < 40 Gy. Similarly, the Dmax for the stomach, small bowel, and duodenum were < 40–45 Gy each [22].

### Anti-PD1 therapy

Patients were treated with anti-PD1 antibodies, including camrelizumab (HengRui Medicine [Jiangsu, China] Co. Ltd.), toripalimab (Junshi Biosciences [Shanghai, China] Co. Ltd), sintilimab (Innovent Biologics [Suzhou, China] Co. Ltd.), or tislelizumab (BeiGene Biosciences [Shanghai, China] Co. Ltd), as concurrent or sequenced therapy in the RT + PD1 group. Patients received anti-PD1 intravenously every three weeks until disease progression, intolerable toxicity, or patient withdrawal. The method of injection, dose, and duration of the anti-PD1 were as recommended by the manufacturer.

### Evaluation of liver hepatic metrics and dosimetric parameters

All patients underwent a CT and/or magnetic resonance imaging (MRI) within one month before the initiation of RT and every 2–3 months after RT to evaluate the hepatic toxicity and tumor response. RIHT was assessed based on the CP scoring system and common toxicity criteria for adverse events (version 5.0) within three months after completion of the RT. The CP score (CP ≥ 1 or CP ≥ 2) is recognized as an effective system for evaluating RIHT [23]. RILD was categorized into two types: classic RILD (cRILD) and non-classic RILD (ncRILD), within three months after completion of the RT. RILD resulted in anicteric hepatomegaly and ascites, an alkaline phosphatase (ALP) level at least twice the upper normal or baseline value (cRILD), an increase in the CP score by two or more, or an increase in alanine aminotransferase (ALT) or aspartate aminotransferase (AST) levels ≥ five times the upper limit of the normal or baseline value (ncRILD) in the absence of tumor progression and/or HBV reactivation (a 10-fold or greater increase in HBV DNA levels) [15, 24, 25].

Dosimetric parameters, including the GTV, normal liver volume (Vliver), mean dose to the normal liver (Dmean), and percentage of normal liver volume receiving > x Gy radiation (Vx, x = 5, 7.5, 10, 15, 20, 25, 30, or 35) were analyzed using DVH [23]. The Vliver was calculated by subtracting the lesion volume from the total liver volume.

### Statistics

For patients in the RT + PD1 and RT groups, we adopted a 1:1 propensity score matching (PSM) method to minimize between-group heterogeneity and selection bias using a logistic regression model. The propensity score for the study included the following: age, sex, hepatitis B virus infection (HBV), CP grade, alpha-fetoprotein, tumor number, max tumor size, interventional therapy, hepatectomy, ablation, prothrombin time (PT), and Dmean. The clinical and dosimetric parameters were estimated using continuous or categorical variables. The chi-squared test (Fisher’s exact test), Student’s *t*-test, and Wilcoxon test were performed to compare the clinical, dosimetric, and hepatotoxicity between patients with RT or RT + PD1.

This study included 96 patients with HCC as factors for ncRILD, which were analyzed using a logistic regression model for univariate (p < 0.1) analysis. The Spearman rank test was used to analyze the correlations between the clinical and dosimetric parameters and that among the various dosimetric parameters (p < 0.2). The nomogram model was generated using the risk factors affecting ncRILD by multivariable logistic regression and assessed using the area under the ROC (AUROC) curves and calibration curve (with 1000 bootstrap resamples). We used R version 4.0.5 (http://www.r-project.org/) and SPSS® version 25.0 software (SPSS, Inc., Chicago, IL, USA) to analyze the data.

## Results

### Patient characteristics and follow-up data

Of the 96 patients, 30 treated with RT + PD1 were matched to 66 treated with RT using PSM. The baseline characteristics, including clinical data and dosimetric factors, were not significantly different between the two groups after PSM (Table 1). In the RT + PD1 group, 10, 17, and 3 patients received anti-PD1 before the first fraction of RT, during RT, and after the last RT fraction, respectively. The patients received a median of five (range: 1–22) cycles of anti-PD1; 20, 2, 2, and 4 patients received camrelizumab, toripalimab, sintilimab, and tislelizumab, respectively.

**Table 1:** Patient baseline demographic and clinical characteristics

Variables	Before PSM			After PSM		
	RT + PD1, n = 30(%)	RT, n = 66(%)	*P* value	RT + PD1, n = 30(%)	RT, n = 30(%)	*P* value
Gender, male	30 (100.0)	58 (87.9)	0.111	30 (100.0)	30 (100.0)	NA
Age, year	54.9 ± 11.2	55.7 ± 11.6	0.750	54.9 ± 11.2	55.8 ± 9.4	0.737
Hepatitis B virus infection, present	28 (93.3)	45 (68.2)	0.016	28 (93.3)	27 (90.0)	1.000*
Hepatitis C virus infection, present	1 (3.3)	0 (0.0)	0.313*	1 (3.3)	0 (0.0)	1.000*
Cirrhosis, present	14 (46.7)	44 (66.7)	0.103	14 (46.7)	20 (66.7)	0.193
ECOG PS			0.781			1.000
0	16 (53.3)	36 (54.6)		16 (53.3)	16 (53.3)	
1	14 (46.7)	30 (45.4)		14 (46.7)	14 (46.7)	
Total bilirubin, µmol/L	13.4 (10.8, 18.0)	13.4 (10.1, 19.9)	0.740	13.4 (10.8, 18.0)	14.9 (10.2, 21.2)	0.971
Albumin, g/L	34.7 ± 4.6	35.0 ± 4.0	0.800	34.7 ± 4.6	34.8 ± 4.2	0.947
Aspartate aminotransferase, U/L	38.0 (33.3, 70.8)	47.0 (32.3, 65.0)	0.693	38.0 (33.3, 70.8)	47.0 (32.5, 63.8)	0.751
Alanine aminotransferase, U/L	34.5 (23.5, 58.0)	32.5 (19.3, 49.8	0.217	34.5 (23.5, 58.0)	36.0 (19.7, 53.8)	0.535
Alkaline phosphatase, U/L	107.5 (75.0, 186.3)	107.0 (79.5, 163.3)	0.890	107.5 (75.0, 186.3)	94.5 (73.8, 166.3)	0.615
Prothrombin time, sec	12.2 (11.7, 12.7)	12.8 (12.0, 13.7)	0.037	12.2 (11.7, 12.7)	12.8 (11.7, 13.1)	0.378
Child-Pugh grade			0.756			0.505
A	23 (76.7)	54 (81.8)		23 (82.1)	26 (86.7)	
B	7 (23.3)	12 (18.2)		7 (23.3)	4 (13.3)	
ALBI score	-2.189 + 0.412	-2.224 + 0.368	0.678	-2.189 + 0.412	-2.200 + 0.379	0.917
ALBI grade			0.622			0.470
1	6 (20.0)	9 (13.6)		6 (20.0)	3 (10.0)	
2/3	24 (80.0)	57 (86.4)		24 (80.0)	27 (90.0)	
Alpha fetoprotein, ≥ 400 ng/ml	15 (50.0)	20 (30.3)	0.103	15 (50.0)	11 (36.7)	0.435
Max tumor size, cm	6.0 (4.7, 7.6)	7.6 (5.4, 11.0)	0.021	6.0 (4.7, 7.6)	6.9 (3.9, 9.6)	0.455
Tumor number ≥ 4	15 (50.0)	31 (47.0)	0.956	15 (50.0)	15 (50.0)	1.000
Macrovascular invasion, present	19 (63.3)	42 (63.6)	1.000	19 (63.3)	17 (56.7)	0.792
BCLC stage			0.427			0.505
A/B	4 (13.3)	15 (22.7)		4 (13.3)	7 (23.3)	
C	26 (86.7)	51 (77.3)		26 (86.7)	23 (76.7)	
Gross tumor volume, cc	188.8 (100.8, 575.0)	383.0 (109.8, 800.5)	0.177	188.8 (100.8, 575.0)	371.5 (74.3, 732.5)	0.554
Normal liver volume, cc	985.5 ± 208.2	987.4 ± 331.3	0.977	985.5 ± 208.2	984.7 ± 298.1	0.990
Mean dose to the normal liver, Gy	13.0 (10.3, 16.9)	16.7 (13.6, 20.9)	0.006	13.0 (10.3, 16.9)	15.1 (12.5, 18.6)	0.137
EQD2^2^, Gy	61.9 (60.0, 75.0)	66.6 (54.4, 75.0)	0.340	61.9 (60.0, 75.0)	64.9 (56.6, 75.0)	0.638
V5, %	61.8 ± 12.9	67.4 ± 18.0	0.127	61.8 ± 12.9	66.2 ± 17.5	0.277
V7.5, %	47.4 (42.8, 58.4)	54.9 (40.3, 70.3)	0.379	47.4 (42.8, 58.4)	52.3 (37.6, 68.7)	0.626
V10, %	39.6 (33.0, 53.7)	46.3 (34.0, 63.5)	0.145	39.6 (33.0, 53.7)	43.3 (33.9, 58.9)	0.540
V15, %	30.5 (21.9, 37.4)	36.3 (25.6, 51.4)	0.054	30.5 (21.9, 37.4)	34.1 (24.5, 46.2)	0.308
V20, %	24.9 (14.8, 31.6)	30.3 (20.2, 42.4)	0.054	24.9 (14.8, 31.6)	29.4 (18.9, 39.4)	0.337
V25, %	19.3 (10.8, 26.3)	24.6 (16.9, 36.6)	0.044	19.3 (10.8, 26.3)	23.0 (14.1, 34.4)	0.322
V30, %	14.6 (7.5, 21.5)	20.0 (14.7, 30.0)	0.032	14.6 (7.5, 21.5)	19.7 (11.5, 27.7)	0.363
V35, %	10.1 (4.6, 17.9)	16.6 (11.5, 24.6)	0.005	10.1 (4.6, 17.9)	15.4 (9.8, 21.5)	0.120
Prior therapy						
Interventional therapy	21 (70.0)	43 (65.2)	0.815	21 (70.0)	20 (66.7)	1.000
Hepatectomy	15 (50.0)	23 (34.9)	0.237	15 (50.0)	12 (40.0)	0.604
Ablation	8 (26.7)	7 (10.6)	0.088	8 (26.7)	5 (16.7)	0.531

* Fisher’s exact test

ALBI, albumin-bilirubin scores; BCLC, Barcelona Clinic Liver Cancer; ECOG PS, Eastern Cooperative Oncology Group-performance status; EQD2, equivalent dose in 2‑Gy fractions; ^2^, using LQ model, α/β = 2 Gy; PD1, the monoclonal antibody against programmed cell death 1; PSM, propensity score matching; RT, radiotherapy; Vx, the percentage of normal liver volume receiving > x Gy radiation (x = 5, 7.5, 10, 15, 20, 25, 30, and 35, respectively)

### Evaluation and incidence of RIHT

Five patients with liver disease were excluded because of tumor progression and HBV reactivation. The incidence of RIHT in the two groups before and after PSM is summarized in Tables 2 and 3. Among the 96 evaluable patients, 17.7%, 39.6%, 14.6%, 5.2%, 3.1%, and 1.0% experienced ncRILD, CP score ≥ 1, CP score ≥ 2, increased AST grade 3, increased ALT grade 3, and increased ALP grade 2 within three months after completion of the RT, respectively. The incidence of ncRILD before PSM showed in Supplemental Fig. 1a. No grade 4/5 hepatotoxicity was observed in any metric, and no grade 3 hepatotoxicity was observed in the metrics of increased ALP, increased total bilirubin, or decreased albumin. None of the patients developed cRILD. Before PSM, increased AST ≥ grade 1 was more frequent in the RT + PD1 group than in the RT group (66.7% vs. 37.9%, p = 0.016), while increased total bilirubin ≥ grade 1 was more frequent in the RT group than in the RT + PD1 group (57.6% vs. 33.3%, p = 0.048). There were no differences in other hepatotoxicity parameters, including ncRILD, CP score ≥ 1, CP score ≥ 2, increased AST ≥ grade 2, increased AST grade 3, increased ALT ≥ grade 1, increased ALT ≥ grade 2, increased ALT grade 3, increased ALP ≥ grade 1, increased ALP grade 2, increased total bilirubin grade 2, decreased albumin ≥ grade 1, and decreased albumin grade 2 (Table 2). Among the 60 evaluable patients after PSM, 23.3%, 38.3%, 18.3%, 5.0%, 5.0%, and 1.7% experienced ncRILD, CP score ≥ 1, CP score ≥ 2, increased AST grade 3, increased ALT grade 3, and increased ALP grade 2 within three months after completion of the RT, respectively. The incidence of ncRILD after PSM showed in Supplemental Fig. 1b. Increased AST ≥ grade 1 occurred more frequently in the RT + PD1 group (p = 0.020) than in the RT group, while there were no significant differences in the other hepatotoxicity parameters after PSM between the two groups (Table 3).

**Table 2:** Post-treatment Hepatotoxicity Metrics before PSM.

Hepatotoxicity Metrics	RT + PD1, n = 30 (%)	RT, n = 49 (%)	*P* value
Increased AST, ≥grade1	20 (66.7)	25 (37.9)	0.016
Increased AST, ≥grade2	1 (3.3)	6 (9.1)	0.428*
Increased AST, grade3	1 (3.3)	4 (6.1)	1.000*
Increased ALT, ≥grade1	13 (43.3)	20 (30.3)	0.311
Increased ALT, ≥grade2	2 (6.7)	5 (7.6)	1.000*
Increased ALT, grade3	0 (0.0)	3 (4.6)	0.550
Increased ALP, ≥grade1	4 (13.3)	15 (22.7)	0.427
Increased ALP, grade2	1 (3.3)	0 (0.0)	0.313*
Increased total bilirubin, ≥grade1	10 (33.3)	38 (57.6)	0.048
Increased total bilirubin, grade2	3 (10.0)	7 (10.6)	1.000
Decreased albumin, ≥grade1	19 (63.3)	27 (10.9)	0.069
Decreased albumin, grade2	9 (30.0)	10 (15.2)	0.157
Increased Child-Pugh score, ≥1	12 (40.0)	26 (39.4)	1.000
Increased Child-Pugh score, ≥2	6 (20.0)	8 (12.1)	0.483
Radiation-induced liver disease	7 (23.3)	10 (15.2)	0.493

* Fisher’s exact test

ALP, alkaline phosphatase; ALT, alanine aminotransferase; AST, aspartate aminotransferase; PD1, the monoclonal antibody against programmed cell death 1; PSM, propensity score matching; RT, radiotherapy

**Table 3:** Post-treatment Hepatotoxicity Metrics after PSM.

Hepatotoxicity Metrics	RT + PD1, n = 30 (%)	RT, n = 30 (%)	*P* value
Increased AST, ≥grade1	20 (66.7)	10 (40.0)	0.020
Increased AST, ≥grade2	1 (3.3)	3 (10.0)	0.612*
Increased AST, grade3	1 (3.3)	2 (6.7)	1.000*
Increased ALT, ≥grade1	13 (43.3)	7 (23.3)	0.171
Increased ALT, ≥grade2	2 (6.7)	3 (10.0)	1.000*
Increased ALT, grade3	0 (0.0)	3 (10.0)	0.237*
Increased ALP, ≥grade1	4 (13.3)	5 (16.7)	1.000*
Increased ALP, grade2	1 (3.3)	0 (0.0)	1.000*
Increased total bilirubin, ≥grade1	10 (33.3)	16 (46.7)	0.429
Increased total bilirubin, grade2	3 (10.0)	3 (10.0)	1.000*
Decreased albumin, ≥grade1	19 (63.3)	11 (36.7)	0.071
Decreased albumin, grade2	9 (30.0)	5 (16.7)	0.360
Increased Child-Pugh score, ≥1	12 (40.0)	11 (36.7)	1.000
Increased Child-Pugh score, ≥2	6 (20.0)	5 (16.7)	1.000
Radiation-induced liver disease	7 (23.3)	7 (23.3)	1.000

* Fisher’s exact test

ALP, alkaline phosphatase; ALT, alanine aminotransferase; AST, aspartate aminotransferase; PD1, the monoclonal antibody against programmed cell death 1; PSM, propensity score matching; RT, radiotherapy

### Prognostic factors for ncRILD

Univariate analyses of all patients after PSM were performed for the clinical and dosimetric factors of ncRILD, as shown in Table 4. The absolute Spearman’s Rho values close to 1 of the dosimetric parameters showed that the two parameters were highly correlated (Supplemental Fig. 2). To avoid overfitting, only a dosimetric risk factor of V25 was included in the model. Optimal predictors, including V25, tumor number, age, and PT, were significantly associated with ncRILD (Table 4). Univariate analyses before PSM were performed for the clinical and dosimetric factors of ncRILD, as shown in Supplemental Table 1. The tumor number and Vliver were significantly associated with ncRILD (Supplemental Table 1).

**Table 4:** Univariate analysis of parameters associated with the risk of ncRILD after PSM (n = 60)

Characteristics	Univariable analysis	
	OR (95%CI)	*P* value
RT + PD1 vs. RT	1.000 (0.302–3.309)	1.000
Gender, male vs. female	NA	NA
Age (year)	1.083 (1.010–1.161)	0.025
Hepatitis B virus infection, positive vs. negative	0.419 (0.063–2.799)	0.369
Hepatitis C virus infection, positive vs. negative	0 (0-Inf)	0.992
Cirrhosis, yes vs. no	1.026 (0.306–3.434)	0.967
ECOG PS, 0 vs. 1	0.818 (0.245–2.734)	0.744
Total bilirubin (µmol/L)	0.998 (0.921–1.082)	0.963
Albumin (g/L)	0.980 (0.855–1.123)	0.769
Aspartate aminotransferase (U/L)	1.002 (0.980–1.024)	0.862
Alanine aminotransferase (U/L)	1.000 (0.985–1.015)	0.995
Alkaline phosphatase (U/L)	1.000 (0.993–1.007)	0.979
Prothrombin time (sec)	1.769 (1.019–3.072)	0.043
Child-Pugh grade, A vs. B	0.685 (0.130–3.621)	0.656
ALBI score	1.322 (0.290–6.017)	0.718
ALBI grade, 1 vs. 2/3	2.737 (0.312–24.021)	0.364
Alpha fetoprotein (ng/ml) ≥ 400 vs. <400	0.661 (0.192–2.280)	0.513
Max tumor size (cm)	1.078 (0.944–1.231)	0.265
Tumor number ≥ 4 vs. <4	3.250 (0.888–11.899)	0.075
Macrovascular invasion, yes vs. no	1.267 (0.366–4.381)	0.709
BCLC stage A/B vs. C	1.459 (0.276–7.713)	0.656
Gross tumor volume (cc)	1.000 (0.999–1.001)	0.857
Normal liver volume (cc)	0.998 (0.996–1.001)	0.133
Mean dose to the normal liver (Gy)	1.000 (0.999–1.001)	0.715
EQD2^2^ (Gy)	0.989 (0.946–1.033)	0.617
V5 (%)	1.024 (0.984–1.065)	0.237
V7.5 (%)	1.019 (0.983–1.056)	0.301
V10 (%)	1.023 (0.986–1.061)	0.229
V15 (%)	1.031 (0.990–1.073)	0.142
V20 (%)	1.037 (0.991–1.086)	0.115
V25 (%)	1.045 (0.994–1.098)	0.084
V30 (%)	1.050 (0.992–1.111)	0.093
V35 (%)	1.056 (0.987–1.130)	0.111
Interventional therapy, yes vs. no	0.525 (0.152–1.811)	0.308
Hepatectomy, yes vs. no	0.400 (0.109–1.461)	0.166
Ablation, yes vs. no	0.530 (0.103–2.742)	0.449

ALBI, albumin-bilirubin scores; BCLC, Barcelona Clinic Liver Cancer; ECOG PS, Eastern Cooperative Oncology Group-performance status; EQD2, equivalent dose in 2‑Gy fractions; ^2^, using LQ model, α/β = 2 Gy; ncRILD, non-classic radiation-induced liver disease; PD1, the monoclonal antibody against programmed cell death 1; PSM, propensity score matching; RT, radiotherapy; Vx, the percentage of normal liver volume receiving > x Gy radiation (x = 5, 7.5, 10, 15, 20, 25, 30, and 35, respectively)

### Nomogram model

**Fig. 2:**
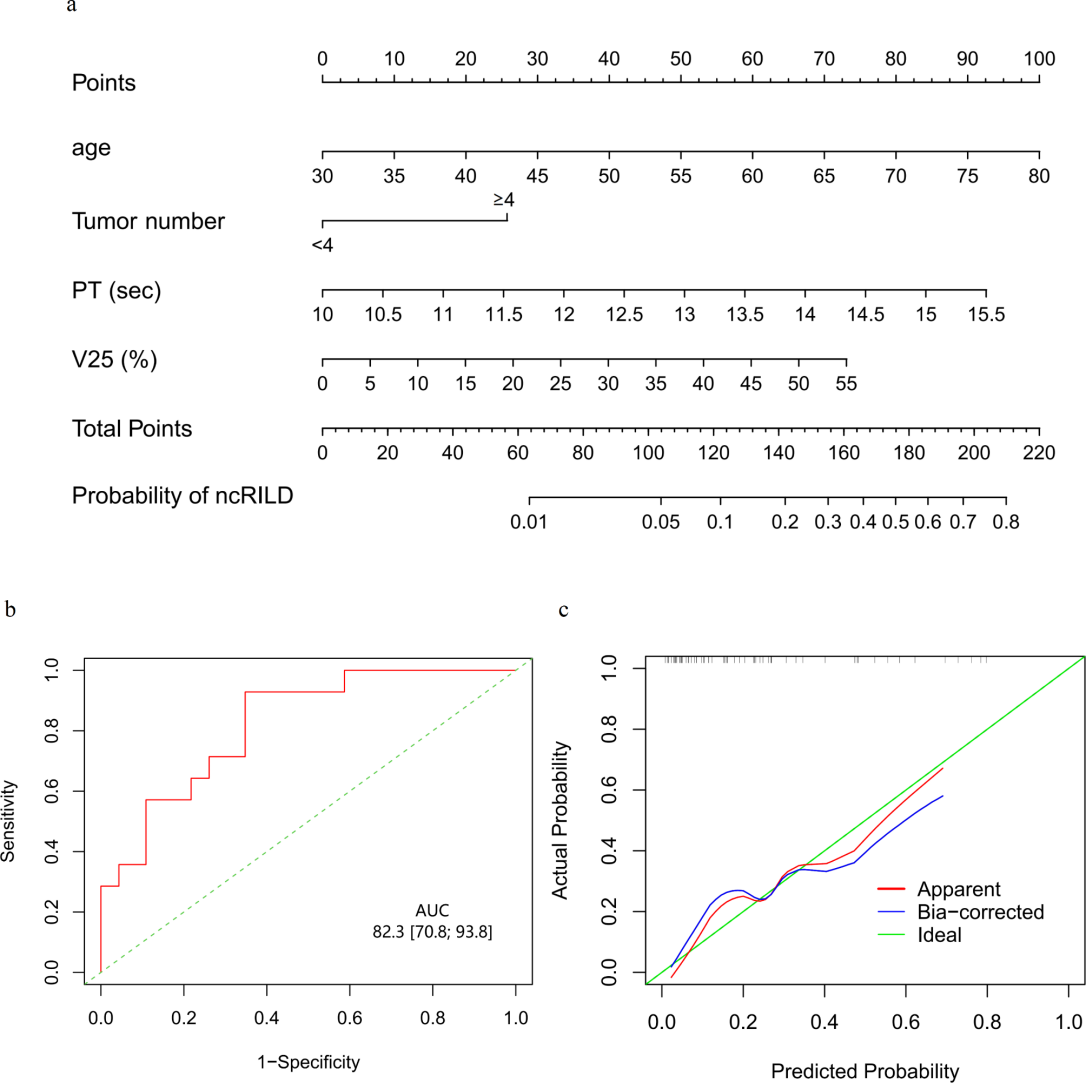
Model prediction and evaluation for ncRILD. (a) Nomogram based on V25, tumor number, age, and PT for ncRILD prediction. The total score for each patient is used to predict the probability of ncRILD. (b) Receiver operating curve curves of the nomogram to predict ncRILD. (c) Calibration curves for ncRILD nomogram prediction. AUC, the area under the curve; ncRILD, non-classic radiation-induced liver disease; PT, prothrombin time; V25, the percentage of normal liver volume receiving > 25 Gy radiation

A nomogram model in the matched cohort was integrated based on multivariable logistic regression (Fig. 2a). The AUROC (0.823, 95% CI, 0.708–0.938) was used to evaluate the prediction of ncRILD (Fig. 2b), and a calibration curve showed a good predictive ability for ncRILD (Fig. 2c).

## Discussion

In recent years, the combination of RT with immunotherapy has received close attention for HCC. RT can enhance antigen presentation and tumor immunogenicity for tumor phenotype modulation, improving the efficacy of cancer immunotherapy [26]. Our previous studies suggested that RT combined with immunotherapy as a novel treatment strategy in patients with HCC showed promising efficacy and acceptable safety and may, therefore, be a promising therapeutic strategy for patients with HCC [13, 27]. RIHT remains a major challenge in patients with HCC undergoing liver irradiation, particularly RILD, which is a serious treatment-related complication [28, 29]. HCC patients receiving anti-PD1 can experience hepatic injury, such as elevation of transaminase or blood bilirubin [18, 19]. To the best of our knowledge, few studies to date have compared the effect of RT plus anti-PD1 versus RT alone on RIHT in patients with HCC. The present study showed that patients who received RT combined with anti-PD1 had a comparable incidence of hepatotoxicity as those who received RT alone before and after PSM. Our findings demonstrated RT plus anti-PD1 may not increase the risk of RIHT over that of RT alone among patients with HCC.

The incidence of hepatotoxicity in the present RT group is similar to that in the literature [25, 30]. Chapman et al. [25] reported that 48%, 25%, 10%, 17%, 13%, 2%, 6%, and 2% of patients with primary liver malignancies who received 30–50 Gy in five fractions with SBRT had at least a CP score increase of 1, CP score increase of 2, total bilirubin of G2, AST of G2, ALT of G2, ALP of G2, AST of G3, and ALT of G3, respectively. In a prospective study using SBRT (39–50 Gy in 3–5 fractions), an increase in CP score ≥ 1 and CP score ≥ 2 was observed in 14.3% and 9.4%, respectively, of 85 patients at three months and in 19.0%, and 11.8%, respectively, of 85 patients at six months. There was no observed cRILD or ncRILD (elevated ALT or AST) [23]. In addition, Jun et al. [31] reported that the incidence of RILD (elevated liver transaminases ≥ grade 3 or CP ≥ 2) was 24.7% among patients with HCC treated with SBRT using 40–60 Gy in 3–5 fractions. In summary, the hepatotoxicity when using RT to treat patients with HCC are acceptable.

A case series of five patients with unresectable HCC who were treated with SBRT followed by anti-PD1 showed that none of the patients developed classic RILD or a CP score ≥ 2. There were 1, 2, and 2 patients who had G1 elevation in AST, G1 elevation in ALT, and G2 elevation in AST/ALT, respectively [12]. However, the number of patients treated with RT combined with anti-PD1 in the study was relatively small. Moreover, in a phase II trial of 21 patients with unresectable HCC treated with combined RT and camrelizumab (an anti-PD1), grade 1–2 adverse events comprised increased AST in 11 patients (52.4%), increased ALT in 10 (47.6%), increased blood bilirubin in 4 (19.1%), and decreased albumin in 11 (52.4%) [13]. These studies showed that the treatment toxicities were manageable in patients with HCC treated with RT + PD1. Similarly, only one patient (3.3%) who received RT combined with anti-PD1 experienced increased AST grade 3, and no other grade 3–5 hepatotoxicity was observed in this study. The hepatotoxicity in the RT + PD1 group did not differ from that in the RT group except for increased AST ≥ grade 1 and increased total bilirubin before PSM and decreased albumin ≥ grade 1 after PSM; these toxicities were mild and manageable. Additionally, the rates of RILD did not differ between the RT and RT + PD1 groups (incidence of 23.3% for both, p = 1.000). Thus, our study showed that the combination of RT with anti-PD1 for patients with HCC was feasible and that its hepatotoxicity was acceptable, although prospective studies are required to improve its safety for further study.

Notably, accurate prediction of RT toxicity in patients with HCC will assist with achieving optimal RT planning, which may help physicians choose the best therapeutic regimen. However, the predictors of hepatotoxicity are not well established. In the present study, cRILD was not observed. Therefore, the relatively serious hepatic toxicity, described as ncRILD, was selected to analyze the prognostic factors for patients with HCC [32]. The results showed that treatment with RT alone or combined with anti-PD1 was not correlated with ncRILD. Several dose-volumetric factors are significantly associated with RILD [15, 33]. In a study of patients who received three-dimensional conformal radiation therapy with a radiation dose of 38–68 Gy and a fraction size of 4–6 Gy, a V25 of 35% showed statistical significance as liver radiation tolerance for RILD. Age, tumor number, and PT were found to be optimal predictors for ncRILD to construct an effective model. Moreover, the tumor number and PT were the most significant factors associated with ncRILD for patients with Child–Pugh grade B with HCC after IMRT [34]. According to the model, the probability of ncRILD was relatively low for patients with lower scores, which predicts the safety of RT. Therefore, in the era of precision oncology, our results may make an important contribution to RT treatment strategies for patients with HCC.

This study had several limitations. First, this study was retrospective, although PSM was used to balance the differences between the two groups. Second, this was a single-center study with a small sample size. Third, the types and schedules of anti-PD1 used for the treatment were heterogeneous, although the best RT / anti-PD-1 schedule, RT dose, fractionation scheme has not been specified yet [35]. Fourth, large number of patients lost lab test (n = 21) may resulted in bias, yet the clinical data of enrolled patients is complete, and we observed that the incidence of RIHT in patients with HCC treated with RT plus anti-PD1 was acceptable and similar to that of patients treated with RT alone. In addition, our study lacks independent validation. Multi-center and prospective studies are required to confirm these findings.

## Conclusions

The results of this study indicate that the incidence of RIHT in patients with HCC treated with RT plus anti-PD1 was acceptable and similar to that of patients treated with RT alone. A nomogram based on V25, tumor number, age, and pre-PT, which are useful predictors of ncRILD, can help with delivering personalized therapy for patients with HCC.

### Electronic supplementary material

Below is the link to the electronic supplementary material.

Supplemental Fig. 1. The cumulative incidence of ncRILD before (a) and after (b) PSM. ncRILD, non-classic radiation-induced liver disease; PSM, propensity score matching.

Supplemental Fig. 2. Spearman’s rank correlation test between clinical and dosimetric parameters. ALP, alpha-fetoprotein; ALBI, albumin-bilirubin scores; ALT, alanine aminotransferase; ALP, alkaline phosphatase; AST, aspartate aminotransferase; BCLC, Barcelona Clinic Liver Cancer; CP, Child–Pugh; Dmean, mean dose to the normal liver; ECOG PS, Eastern Cooperative Oncology Group performance status; EQD2, equivalent dose in 2‑Gy fractions; 2, using LQ model, α/β = 2 Gy; GTV, gross tumor volume; HBV, chronic hepatitis B virus infection; HCC, hepatocellular carcinoma; MVI, macrovascular invasion; anti-PD1, monoclonal antibody against programmed cell death 1; RT, radiotherapy; Vliver, normal liver volume; PT, prothrombin time; Vx, the percentage of normal liver volume receiving > x Gy radiation (x = 5, 7.5, 10, 15, 20, 25, 30, or 35).

Supplementary Material 3

## Data Availability

The data underlying this article will be shared on reasonable request to the corresponding author.

## Abbreviations

AUROC: Area under the receiver operating characteristic
AST: Aspartate aminotransferase
ALT: Alanine aminotransferase
ALP: Alkaline phosphatase
BCLC: Barcelona Clinic Liver Cancer
cRILD: Classic radiation-induced liver disease
CP: ChildPugh
CT: Computed tomography
Dmean: Mean dose to the normal liver
DVH: Dose‒volume histogram
GTV: Gross tumor volume
HBV: Chronic hepatitis B virus infection
HCC: Hepatocellular carcinoma
IMRT: Intensity-modulated radiation therapy
ncRILD: Non-classic radiation-induced liver disease
OARs: Organs at risk
PTV: Planning target volume
PSM: Propensity score matching
RIHT: Radiation-induced hepatic toxicity
RILD: Radiation-induced liver disease
anti-PD1: Antibodies against programmed cell death protein 1
RT: Radiotherapy
SBRT: Stereotactic body radiation therapy
Vliver: Normal liver volume
VMAT: Volumetric-modulated arc therapy
