# Supplementary figures and images for: Radiotherapy plus anti-PD1 versus radiotherapy for hepatic toxicity in patients with hepatocellular carcinoma
Source: Radiat Oncol. 2023 Aug 4;18:129. doi: 10.1186/s13014-023-02309-1 (PMC10403970; doi:10.1186/s13014-023-02309-1)

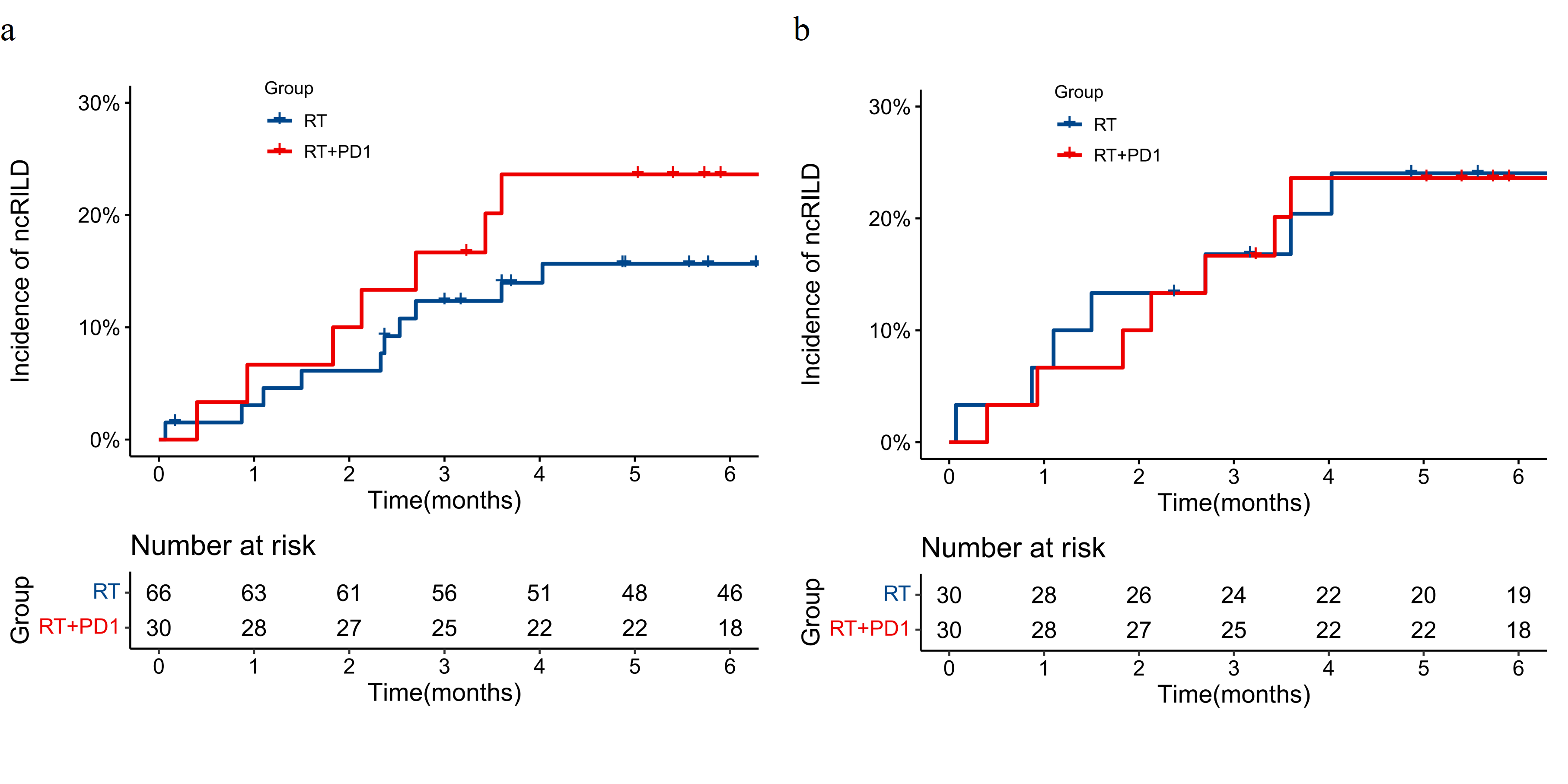

Supplement: Supplementary file 1 — Supplemental Fig. 1. The cumulative incidence of ncRILD before (a) and after (b) PSM. ncRILD, non-classic radiation-induced liver disease; PSM, propensity score matching. [file 13014_2023_2309_MOESM1_ESM.tif]

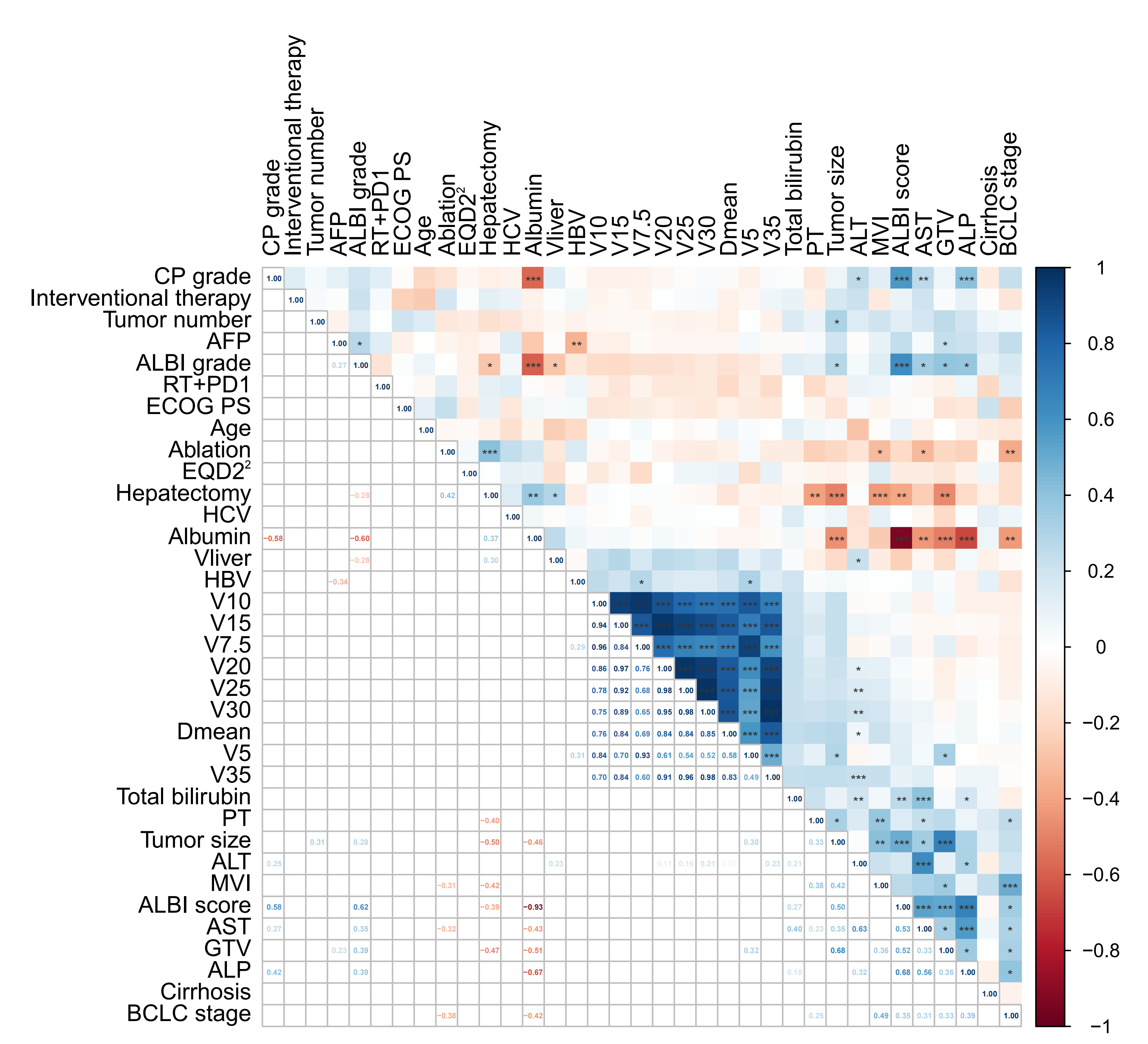

Supplement: Supplementary file 2 — Supplemental Fig. 2. Spearman’s rank correlation test between clinical and dosimetric parameters. ALP, alpha-fetoprotein; ALBI, albumin-bilirubin scores; ALT, alanine aminotransferase; ALP, alkaline phosphatase; AST, aspartate aminotransferase; BCLC, Barcelona Clinic Liver Cancer; CP, Child–Pugh; Dmean, mean dose to the normal liver; ECOG PS, Eastern Cooperative Oncology Group performance status; EQD2, equivalent dose in 2‑Gy fractions; 2, using LQ model, α/β = 2 Gy; GTV, gross tumor volume; HBV, chronic hepatitis B virus infection; HCC, hepatocellular carcinoma; MVI, macrovascular invasion; anti-PD1, monoclonal antibody against programmed cell death 1; RT, radiotherapy; Vliver, normal liver volume; PT, prothrombin time; Vx, the percentage of normal liver volume receiving > x Gy radiation (x = 5, 7.5, 10, 15, 20, 25, 30, or 35). [file 13014_2023_2309_MOESM2_ESM.tif]
