# Supplementary material for: Radiotherapy plus anti-PD1 versus radiotherapy for hepatic toxicity in patients with hepatocellular carcinoma
Source: Radiat Oncol. 2023 Aug 4;18:129. doi: 10.1186/s13014-023-02309-1 (PMC10403970; doi:10.1186/s13014-023-02309-1)
Supplement: Supplementary file 3 — Supplementary Material 3 [file 13014_2023_2309_MOESM3_ESM.docx]

Supplemental table 1. Univariate analysis of parameters associated with the risk of ncRILD before PSM (n=96).

| Characteristics | Univariable analysis | |
| --- | --- | --- |
|  | OR (95%CI) | *P* value |
| RT+PD1 vs. RT | 1.704 (0.578-5.024) | 0.334 |
| Gender, male vs. female | 1.556 (0.179-13.545) | 0.689 |
| Age (year) | 1.027 (0.980-1.076) | 0.266 |
| Hepatitis B virus infection, positive vs. negative | 1.029 (0.300-3.534) | 0.964 |
| Hepatitis C virus infection, positive vs. negative | 0 (0-Inf) | 0.992 |
| Cirrhosis, yes vs. no | 0.689 (0.240-1.979) | 0.489 |
| ECOG PS, 0 vs. 1 | 0.589 (0.198-1.747) | 0.340 |
| Total bilirubin (μmol/L) | 0.974 (0.902-1.053) | 0.510 |
| Albumin (g/L) | 0.972 (0.857-1.101) | 0.653 |
| Aspartate aminotransferase (U/L) | 1.004 (0.987-1.022) | 0.626 |
| Alanine aminotransferase (U/L) | 1.003 (0.990-1.017) | 0.651 |
| Alkaline phosphatase (U/L) | 0.999 (0.992-1.006) | 0.799 |
| Prothrombin time (sec) | 1.307 (0.888-1.923) | 0.174 |
| Child-Pugh grade, A vs. B | 0.844 (0.216-3.295) | 0.807 |
| ALBI score | 1.139 (0.288-4.504) | 0.853 |
| ALBI grade, 1 vs. 2/3 | 3.446 (0.421-28.177) | 0.248 |
| Alpha fetoprotein (ng/ml) ≥400 vs. <400 | 1.275 (0.437-3.718) | 0.656 |
| Max tumor size (cm) | 1.020 (0.903-1.152) | 0.750 |
| Tumor number ≥4 vs. <4 | 3.250 (0.888-11.899) | 0.075 |
| Macrovascular invasion, yes vs. no | 1.267 (0.366-4.381) | 0.507 |
| BCLC stage A/B vs. C | 1.469 (0.471-4.585) | 0.656 |
| Gross tumor volume (cc) | 1.000 (0.999-1.001) | 0.976 |
| Normal liver volume (cc) | 0.998 (0.996-1.000) | 0.042 |
| Mean dose to the normal liver (Gy) | 1.000 (0.999-1.000) | 0.363 |
| EQD2^2^ (Gy) | 1.000 (0.964-1.037) | 0.979 |
| V5 (%) | 1.004 (0.973-1.036) | 0.800 |
| V7.5 (%) | 0.998 (0.969-1.028) | 0.916 |
| V10 (%) | 0.999 (0.970-1.029) | 0.953 |
| V15 (%) | 1.002 (0.970-1.035) | 0.900 |
| V20 (%) | 1.001 (0.967-1.037) | 0.935 |
| V25 (%) | 1.004 (0.968-1.041) | 0.836 |
| V30 (%) | 1.003 (0.964-1.044) | 0.887 |
| V35 (%) | 1.000 (0.957-1.045) | 1.000 |
| Interventional therapy, yes vs. no | 0.661 (0.226-1.940) | 0.451 |
| Hepatectomy, yes vs. no | 0.581 (0.187-1.807) | 0.348 |
| Ablation, yes vs. no | 0.677 (0.138-3.322) | 0.631 |

ALBI, albumin-bilirubin scores; BCLC, Barcelona Clinic Liver Cancer; ECOG PS, Eastern Cooperative Oncology Group-performance status; EQD2, equivalent dose in 2‑Gy fractions; ^2^, using LQ model, α/β = 2Gy; ncRILD, non-classic radiation-induced liver disease; PD1, the monoclonal antibody against programmed cell death 1; PSM, propensity score matching; RT, radiotherapy; Vx, the percentage of normal liver volume receiving > x Gy radiation (x = 5, 7.5, 10, 15, 20, 25, 30, and 35, respectively).
